# Prevalence and Functional Implication of Silent Coronary Artery Disease in Marathon Runners Over 40 Years of Age: The MATCH-40 Study

**DOI:** 10.1016/j.cjco.2020.12.024

**Published:** 2021-01-07

**Authors:** Christopher T. Lee, Skyler E. Eastman, Liane A. Arcinas, Chantal Y. Asselin, David Cheung, Andrew Mayba, Antonia Zhu, Jacek Strzelczyk, Bruce Maycher, Brett Memauri, Iain D.C. Kirkpatrick, Davinder S. Jassal

**Affiliations:** a Institute of Cardiovascular Sciences, St. Boniface Albrechtsen Research Centre, University of Manitoba, Winnipeg, Manitoba, Canada; b Section of Cardiology, Department of Internal Medicine, Max Rady College of Medicine, Rady Faculty of Health Sciences, University of Manitoba, Winnipeg, Manitoba, Canada; c Department of Radiology, Max Rady College of Medicine, Rady Faculty of Health Sciences, University of Manitoba, Winnipeg, Manitoba, Canada

## Abstract

**Background:**

Marathon participation is becoming increasingly popular among individuals ≥40 years of age. Little is known about the prevalence of subclinical coronary artery disease (CAD) and corresponding ischemia in this patient population. The study objectives are: (1) to characterize the prevalence of silent CAD in marathoners ≥ 40 years old using cardiac computed tomography angiography (CCT); and (2) if subclinical CAD was detected, to determine the functional significance of occult lesions by stress echocardiography (SE).

**Methods:**

Marathoners aged ≥ 40 years who completed a full marathon between 2018 and 2019 were recruited to undergo a prospective CCT. Coronary artery stenosis was graded as zero, mild (1%-49%), moderate (50%-69%), or severe (> 70%). All study participants diagnosed with mild-to-severe atherosclerotic CAD on CCT further underwent functional imaging with exercise treadmill SE.

**Results:**

A total of 65 individuals (53 ± 7 years, 65% males, 24 ± 3 kg/m^2^) underwent a prospective CCT within 12 months of marathon completion. Of the total study population, 13 participants (20%) were diagnosed with CAD, of whom 10 (77%) had mild disease, 1 (8%) had moderate disease, and 2 (15%) had severe disease by CCT. Despite the identification of subclinical CAD on CCT, none of the 13 patients had any evidence of inducible ischemia on SE.

**Conclusions:**

This is the first study to incorporate both CCT and SE in the evaluation of subclinical CAD in marathoners ≥40 years old. Although the overall prevalence of anatomic CAD was 20%, there was no evidence of functional ischemia in this highly competitive cohort.

## Résumé

**Contexte:**

Les marathons ont gagné en popularité auprès des individus âgés de 40 ans ou plus. On en sait toutefois peu sur la prévalence de la coronaropathie subclinique et de l’ischémie qui lui est associée dans cette population de patients. L’étude visait à 1) caractériser la prévalence de la coronaropathie silencieuse chez les marathoniens âgés de 40 ans ou plus à l’aide d’une angiographie cardiaque par tomodensitométrie (ACTDM) si une coronaropathie subclinique était détectée, à déterminer l’importance fonctionnelle des lésions occultes par une échocardiographie d’effort (EE).

**Méthodologie:**

Des marathoniens âgés de 40 ans ou plus ayant réalisé un marathon entre 2018 et 2019 ont été recrutés et soumis à une ACTDM prospective. Les sténoses des artères coronaires étaient classées selon une échelle allant de zéro, légère (1 à 49 %), modérée (50 à 69 %) à sévère (> 70 %). Tous les participants à l’étude ayant reçu un diagnostic de coronaropathie athéroscléreuse légère à sévère à la suite de l’ACTDM ont été soumis à une imagerie fonctionnelle avec EE sur tapis roulant.

**Résultats:**

Au total, 65 sujets (53 ± 7 ans, 65 % d’hommes, 24 ± 3 kg/m^2^) ont été soumis à une ACTDM prospective dans un délai de 12 mois à la suite de leur dernier marathon. Dans l’ensemble de la population à l’étude, 13 participants (20 %) ont reçu un diagnostic de coronaropathie; 10 (77 %) présentaient une maladie bénigne, 1 (8 %) présentait une maladie modérée et 2 (15 %) présentaient une maladie sévère selon l’ACTDM. Même si une coronaropathie subclinique a été diagnostiquée lors de l’ACTDM, aucun des 13 patients ne présentait de signe d’ischémie inductible à l’EE.

**Conclusions:**

Il s’agit de la première étude à utiliser l’ACTDM et l’EE pour évaluer la présence d’une coronaropathie chez des marathoniens âgés de 40 ou plus. Même si la prévalence globale de la coronaropathie anatomique était de 20 %, il n’y avait aucun signe d’ischémie fonctionnelle au sein de cette cohorte hautement compétitive.

Marathon running has become progressively more popular over the past 2 decades in North America, particularly among individuals over 40 years of age.^1^ The increase in marathon participation has been driven mainly by the increased public awareness of the perceived health benefits associated with regular physical exercise. With a rising enrolment in marathon running among an older population, an increasing rate of sudden cardiac death (SCD) has been observed in competitive races. These unfortunate cases of SCD have generated concerns regarding the adverse health risks associated with marathon participation, especially given that marathon athletes are typically at their peak state of physical conditioning. A recent study reported that in the United States, 2 in every 100,000 male athletes (mean age 52 years) suffered SCD during a marathon, an incidence that has increased 2-fold in recent years.^2^ Underlying atherosclerotic coronary artery disease (CAD) is among the most common causes of exercised-related death in adult marathoners ≥ 40 years of age.^2^^,^^3^

Given the increasing proportion of individuals older than 40 years interested in participating in marathon running, how do we address their cardiac safety profile before participation in this endurance activity? Noninvasive cardiovascular imaging may be used to identify early subclinical stages of atherosclerotic disease.^4^ Cardiac computed tomography angiography (CCT) is a valuable imaging modality for the detection and risk stratification of CAD. CCT can detect and quantify coronary artery calcification (CAC) and obstructive coronary artery stenosis with a spatial resolution comparable with invasive coronary angiography (ICA).^5^ Furthermore, recent advances in CT technology have minimized the overall radiation dose of a prospective CCT to 1.0 mSV.^6^

In recent years, studies have evaluated CCT in marathon runners ≥ 40 years of age. Collectively, these studies demonstrate a prevalence of CAD of up to 60% using CAC and/or coronary artery stenosis detected on CCT.^7^, ^8^, ^9^, ^10^ Although routine electrocardiogram (ECG) exercise stress testing was employed in 2 of these studies as part of the inclusion criteria, no routine functional imaging studies were performed.^7^^,^^8^ Although exercise testing can diagnose ischemia and provide useful prognostic information, the overall sensitivity and specificity is comparatively low, with a limited capability to localize ischemia.^11^^,^^12^ Conversely, stress echocardiography (SE) is a widely available, cost-effective modality, with higher sensitivity and specificity for detecting underlying ischemic heart disease.^13^ Little is known, however, regarding the role of both CCT and SE for the diagnosis of ischemia in the marathon population.

The objectives of the **Ma**ra**t**hon Coronary **C**omputed Tomography Angiography in **H**ealthy Runners Over **40** (MATCH-40) study were 2-fold: (1) to quantify the prevalence of silent CAD in marathoners aged ≥ 40 in a Canadian population using CCT; and (2) if subclinical CAD was detected, to determine the functional significance of occult lesions by SE.

## Methods

### Study population

A total of 105 individuals ≥ 40 years of age who participated in the full Manitoba Marathons between 2018 and 2019 were approached. The inclusion criteria included age ≥ 40 years and successful completion of the 2018 or 2019 full Manitoba Marathon. The exclusion criteria included symptoms suggestive of CAD and any prior history of CAD including previous percutaneous coronary intervention (PCI) or coronary artery bypass surgery, contraindication to CCT, and/or unwillingness to give informed consent. Baseline demographics including age, height, weight, cardiovascular risk factors, medications, allergies, prior medical or surgical history, prior marathon history, and training regimen were recorded for each study participant. The study protocol was approved by the local institutional review board (University of Manitoba REB: B2018:006). Written informed consent was obtained from all participants.

### Cardiac CT

**Figure 1:**
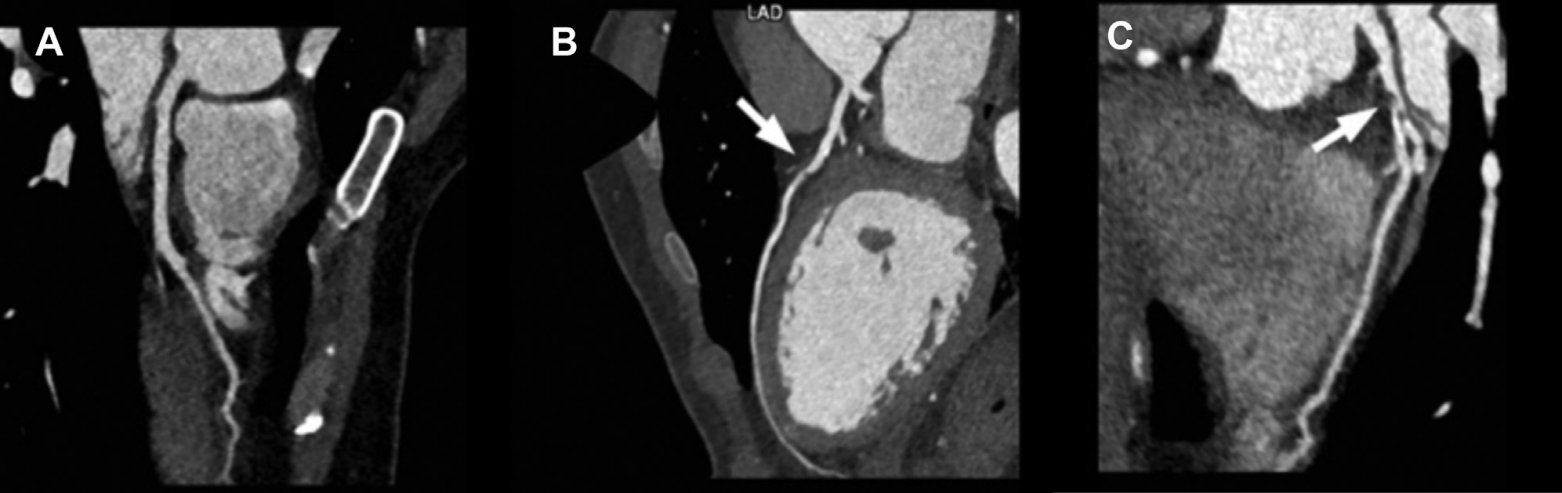
Representative cardiac computed tomography angiography images of the coronary arteries. (**A**) Curved planar reformation (CPR) demonstrating no evidence of coronary artery disease in the left anterior descending artery (LAD). (**B**) CPR demonstrating 50% stenosis of the LAD due to noncalcified plaque. (**C**) Multiplanar reformation demonstrating > 70% stenosis of the LAD due to noncalcified plaque.

All patients underwent CCT imaging using a 128-row dual source multidetector CT (Definition Flash; Siemens Healthineers, Erlangen, Germany). In preparation for the scan, study participants with a resting heart rate (HR) > 60 beats per minute (bpm) received an oral beta blocker (metoprolol up to 150 mg po), unless the systolic blood pressure (BP) was less than 100 mm Hg, or other contraindications existed. All study participants received 0.3 mg of sublingual nitroglycerin before the CCT scan. Either a prospective ECG-gated or high-pitch helical ECG-gated CCT was performed depending on the patient’s baseline HR. CCT data sets on the 128-row dual source CT were obtained using a tube rotation time of 0.28 seconds, 80-120 kV, and 0.6-mm-thick contiguous images through the coronary arteries with a field of view to cover the heart with an ideal cardiac phase coverage of 70%-80%. After a 30-mL timing bolus, each study participant received a total of 72 mL of iohexol (350 mg/mL; Omnipaque 350, GE HealthCare, Milwaukee, WI) followed by 50 mL of a mixture of 40% iohexol/60% normal saline and then 30 mL of normal saline flush; all power was injected at a rate of 6 mL/s. CCT data were analyzed on a 3D workstation equipped with a dedicated cardiac CT software package (syngo.via cardiac applications module; Siemens Healthineers). Source images for the CCTs were analyzed along with multiplanar reformations, curved planar reformations of each coronary artery, and double oblique reformations for each vessel axial to the lumen throughout its course. The presence of coronary atherosclerotic plaque per segment, whether calcified or noncalcified, in addition to severity was determined.^14^, ^15^, ^16^ Coronary artery stenosis was graded as zero, mild (1%-49%), moderate (50%-69%), or severe (> 70%) (Fig. 1).^17^ The CCTs were read by 2 independent level 3 certified experts (D.S.J. and I.D.C.K.) blinded to all demographic data.

### Exercise stress echocardiography

All study participants who were diagnosed with any degree of coronary artery stenosis (mild, moderate, or severe) underwent exercise SE. SE was performed using a General Electric Vivid 7 machine (Milwaukee, IL) following our local protocols guided by the American Society of Echocardiography.^18^ Patients were exercised via a treadmill with manual serial BP readings and continuous 12-lead electrocardiographic monitoring. Overall workload was increased according to the Bruce protocol to a target of 85% maximal predicted HR. At each stage, left ventricular (LV) systolic function and LV regional wall motion abnormalities (RWMA) were recorded using echocardiography. A positive exercise ECG test result was defined as the occurrence of exercise limiting symptoms (including angina, dyspnea, or syncope), fall in systolic BP >20 mm Hg, ST segment depression >2 mm, ST segment elevation > 1 mm, and/or sustained ventricular arrhythmias. A positive SE result was defined as exercise-induced impairment in LV systolic function or LV RWMA. All studies were interpreted by a level 3 echocardiologist (D.S.J.).

### Statistical analysis

The data are summarized as mean ± standard deviation, number (percentage), or median and interquartile range. Paired Student *t* tests were used to compare continuous variables. χ^2^ and Fisher exact tests were applied to compare categorical variables. A *P* value of < 0.05 was considered statistically significant. SAS version 9.4 (SAS Institute Inc, Cary, NC) was used to perform the analyses.

## Results

### Study population

**Figure 2:**
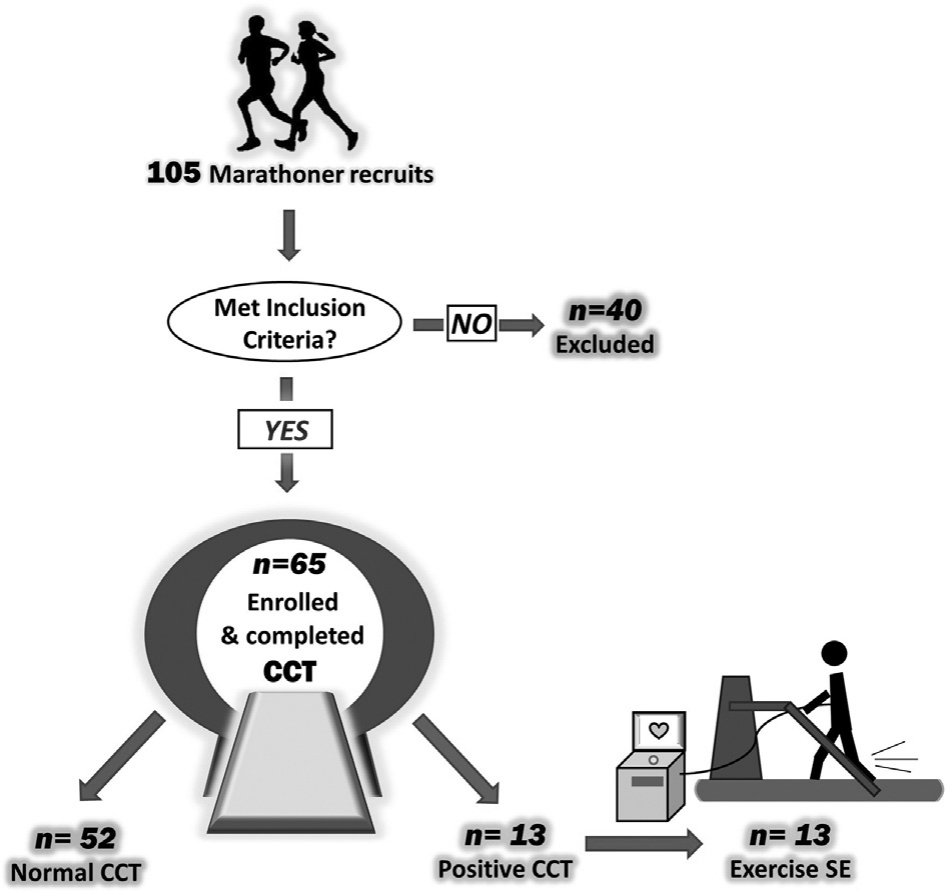
Study design and enrolment. CCT, cardiac computed tomography angiography; SE, stress echocardiography.

A total of 105 individuals ≥ 40 years of age who participated in the Manitoba Marathon between 2018 and 2019 were eligible for the MATCH-40 study. Of the total study population, 65 individuals met inclusion criteria and 40 were excluded (Fig. 2). Baseline demographics including age, sex, body mass index, training experience, marathon experience, and cardiovascular risk factors are presented in Table 1. Of the total study population, there were 23 females (53 ± 8 years) and 42 males (54 ± 7 years).

**Table 1:** Baseline characteristics of the study population (n = 65)

Baseline characteristics	Total population (n = 65)	Males (n = 42)	Females (n = 23)
Age (years ± SD)	53 ± 7	54 ± 7	53 ± 8
BMI (kg/m^2^ ± SD)	24 ± 3	25 ± 3	23 ± 2
Marathon experience			
Lifetime full marathons (n ± SD)	12 ± 14	15 ± 13	8 ± 15
Lifetime half marathons (n ± SD)	18 ± 15	18 ± 16	19 ± 15
Training mileage (miles/wk ± SD)	33 ± 13	36 ± 13	30 ± 13
Marathon time (h:min:s ± SD)	4:31:35 ± 1:19:17	4:31:35 ± 1:19:19	5:31:35 ± 1:19:15
Cardiovascular risk factors			
Known CAD, n (% cohort)	0 (0)	0 (0)	0 (0)
Hypertension, n (% cohort)	5 (8)	2 (5)	3 (13)
Dyslipidemia, n (% cohort)	12 (18)	8 (19)	4 (17)
Diabetes, n (% cohort)	1 (1.5)	1 (2)	0 (0)
Family history of premature CAD, n (% cohort)	10 (15)	6 (14)	4 (17)
Current smoker, n (% cohort)	0 (0)	0 (0)	0 (0)

Data are expressed as mean ± SD.

BMI, body mass index; CAD, coronary artery disease; SD, standard deviation.

### Cardiac CT findings

The mean estimated radiation exposure of all studies was 1.1 ± 0.6 mSV at an average resting HR of 54 ± 10 bpm. A total of 26 individuals (40%) required the administration of an oral beta blocker (metoprolol) before the CCT. Detailed CCT parameter results are summarized in Table 2.

**Table 2:** CCT results of study population (n = 65)

CCT parameters	Total population (n = 65)	Males (n = 42)	Females (n = 23)
Radiation (mSV ± SD)	1.1 ± 0.6	1.3 ± 0.6	0.7 ± 0.4
Resting HR (beats/min ± SD)	54 ± 10	53 ± 8	56 ± 12
Beta blocker administered, n (%)	26 (40)	13 (31)	13 (57)
CCT quality			
Good to excellent, n (%)	61 (94)	40 (95)	21 (91)
Fair, n (%)	4 (6)	2 (5)	2 (9)
Positive CCT, n (%)	13 (20)	10 (24)	3 (13)
Negative CCT, n (%)	52 (80)	32 (76)	20 (87)
Plaque type			
Calcific, n (%)	9 (69)	7 (70)	2 (66)
Noncalcific, n (%)	3 (23)	2 (20)	1 (33)
Mixed, n (%)	1 (8)	1 (10)	0 (0)
Number of atherosclerotic lesions diagnosed by CCT (n)	34	27	7
Mean lesions per positive CCT (n ± SD)	2.6 ± 1.5	2.7 ± 1.5	2.3 ± 1.5

Data are expressed as mean ± SD.

CCT, coronary computed tomography; HR, heart rate; mSV, millisievert; SD, standard deviation.

**Figure 3:**
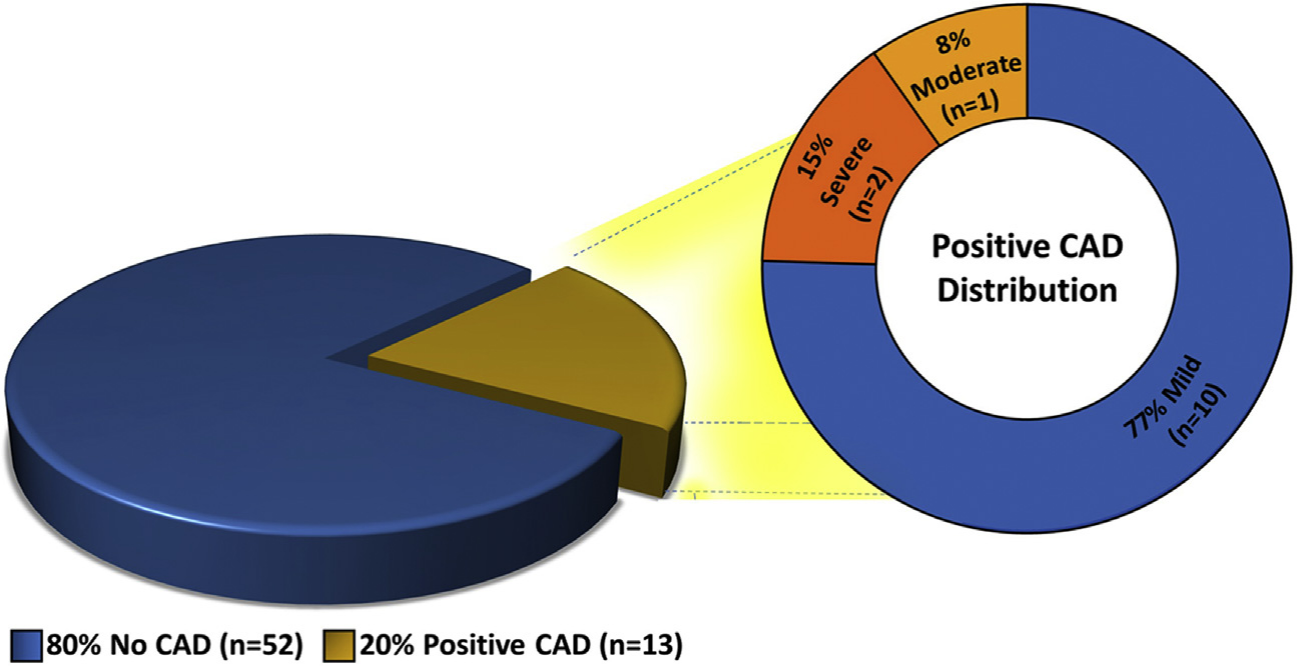
Prevalence and severity of CAD in the study population. CAD, coronary artery disease.

Of the 65 study participants, 52 (80%) had no CAD and 13 (20%) had subclinical CAD as per diagnostic criteria of any degree of coronary artery stenosis (Fig. 3). Of the 13 participants identified with subclinical CAD, 10 (77%) had mild disease, 1 (8%) had moderate disease, and 2 (15%) had severe disease (Fig. 3). Specifically, 3 of 23 females (13%) and 10 of 42 males (24%) were diagnosed with silent CAD. All 3 females with silent CAD had mild CAD (< 50% stenosis). In addition, of the 10 males with silent CAD, 7 had mild CAD (<50% stenosis), 1 had moderate CAD (50%-69% stenosis), and 2 had severe CAD (>70% stenosis). Of the 2 males with severe CAD, 1 study participant had severe diffuse disease of the left anterior descending artery and 1 had a severe focal disease of the right coronary artery. The majority of lesions were calcific (n = 9 [69%]), with a smaller minority of noncalcific (n = 3 [23%]) and mixed lesions (n = 1 [8%]). The mean number of lesions per participants was 2 ± 1.5. Comparing the cohort of these participants diagnosed with silent CAD vs no CAD, the data demonstrate a higher prevalence of premature family history of CAD (23% vs 13%) and dyslipidemia (31% vs 15%) in those individuals with silent CAD, as shown in Table 3. All of the positive CCT participants (n = 13) were initiated on aspirin and a lipid-lowering treatment regimen barring no contraindication.

**Table 3:** Baseline characteristics of patients with silent CAD vs those without silent CAD

Baseline characteristics	Positive CT scans			Negative CT scans		
	Total (n = 13)	Male (n = 10)	Female (n = 3)	Total (n = 52)	Male (n = 32)	Female (n = 20)
Age (y ± SD)	55 ± 10	54 ± 8	61 ± 15	53 ± 7	54 ± 7	52 ± 6
BMI (kg/m^2^ ± SD)	23 ± 3	23 ± 3	21 ± 2	24 ± 2.9	25 ± 3.1	23 ± 1.7
Marathon experience						
Lifetime full marathons (n ± SD)	17 ± 13	21 ± 14	7 ± 5	11 ± 14	13 ± 12	9 ± 16
Lifetime half marathons (n ± SD)	21 ± 18	18 ± 18	32 ± 16	16 ± 15	17 ± 15	17 ± 14
Training mileage (miles/wk ± SD)	33 ± 13	33 ± 13	32 ± 16	34 ± 13	37 ± 13	30 ± 13
Marathon time (h:min:s ± SD)	4:27:39 ± 1:21:16	4:24:39 ± 1:21:18	4:37:42 ± 2:23:05	4:32:34 ± 1:18:18	4:33:33 ± 1:18:18	5:30:34 ± 1:19:16
*Cardiovascular risk factors*						
Known CAD, n (% cohort)	0 (0)	0 (0)	0 (0)	0 (0)	0 (0)	0 (0)
Hypertension, n (% cohort)	0 (0)	0 (0)	0 (0)	5	2	3
Dyslipidemia, n (% cohort)	4 (31)	3 (30)	1 (33)	8 (15)	5 (16)	3 (15)
Diabetes, n (% cohort)	0 (0)	0 (0)	0 (0)	1 (1)	1 (3)	0 (0)
Family history of premature CAD, n (% cohort)	3 (23)	2 (20)	1 (33)	7 (13)	4 (13)	3 (15)
Current smoker, n (% cohort)	0 (0)	0 (0)	0 (0)	0 (0)	0 (0)	0 (0)

Data are expressed as mean ± SD.

BMI, body mass index; CAD, coronary artery disease; CT, computed tomography; SD, standard deviation.

### Stress echocardiography findings

All 13 participants with positive CCTs for any degree of subclinical CAD underwent exercise treadmill SE. The resting HR was 56 ± 8 bpm with a peak HR of 164 ± 10 bpm, reflecting 100 ± 5% maximal predicted HR. The mean total exercise time was 13 ± 3 minutes with a total activity level of 14 ± 3 metabolic equivalent of task (Table 4). No participants experienced cardiovascular symptoms during the stress echocardiograms. Of the 13 participants with positive CCTs for CAD, 4 participants had abnormal ECG changes during exercise. Although 2 individuals demonstrated ST changes suggestive of ischemia and another 2 demonstrated equivocal ST changes, no resting or exercise-induced RWMA were demonstrated on SE. All candidates had normal resting LV ejection fraction at baseline, which augmented normally with exercise. All 13 stress echocardiograms were negative for inducible ischemia. Exercise stress echocardiogram results are summarized in Table 4.

**Table 4:** Stress echocardiogram results from a subset of participants with positive CCT (n = 13)

Exercise stress echocardiogram parameters	Total population (n = 13)	Males (n = 10)	Females (n = 3)
Diagnostic quality (%)	100	100	100
Peak systolic blood pressure (mm Hg ± SD)	198 ± 24	199 ± 26	195 ± 16
Baseline HR (bpm ± SD)	56 ± 8	55 ± 7	59 ± 13
Maximal HR (bpm ± SD)	164 ± 10	165 ± 11	161 ± 9
% Predicted	100 ± 5	100 ± 5	101 ± 7
Exercise time (min ± SD)	13 ± 3	13 ± 2	14 ± 4
Metabolic equivalents (METS ± SD)	14 ± 3	14 ± 3	16 ± 5
ECG results for ischemia			
Positive, n (%)	2 (15.4)	1 (10)	1 (33)
Equivocal, n (%)	2 (15.4)	2 (20)	0 (0)
Negative, n (%)	9 (69.2)	7 (70)	2 (66)
Symptoms during stress, n (%)	0 (0)	0 (0)	0 (0)
Arrhythmias, n (%)	2 (15)	2 (20)	0 (0)
LVEF baseline (%)	> 60	> 60	> 60
LVEF peak exercise (%)	> 60	> 60	> 60
Wall motion at baseline, n (%)	0 (0)	0 (0)	0 (0)
Wall motion at peak exercise, n (%)	0 (0)	0 (0)	0 (0)

Data are expressed as mean ± SD.

bpm, beats per minute; CCT, coronary computed tomography; ECG, electrocardiogram; HR, heart rate; LVEF, left ventricular ejection fraction; METS, metabolic equivalent of task; SD, standard deviation.

## Discussion

As the aging population in North America will increase dramatically over the next 25 years, the proportion of individuals ≥ 40 years of age participating in positive healthy behaviours and increased physical activity will rise accordingly. In the current MATCH-40 study, the overall prevalence of silent CAD was 20%, suggesting that 1 in 5 marathoners over the age of 40 may have subclinical CAD. Despite the presence of anatomic CAD on CCT in this highly competitive cohort, there was no evidence of functional ischemia detected on SE. Whether primary preventative medical therapy with aspirin and lipid-lowering agents may improve positive vessel remodelling and decrease the overall burden of SCD in these individuals with subclinical CAD as confirmed on CCT requires further study.

Interventional coronary angiography remains the gold standard for the diagnosis and characterization of CAD. However, given its invasive nature, ICA is generally reserved as a confirmatory test for individuals whose symptoms and functional tests suggest a potential benefit for PCI.^19^ In recent years, CCT has allowed for comparable anatomic assessment of CAD as an alternative noninvasive imaging modality, which allows for earlier detection of CAD.^19^ The prevalence of subclinical CAD in the general asymptomatic population was evaluated by Lee et al.,^20^ whereby 4320 asymptomatic volunteers aged 35-75 years underwent CCT. In this study, where a positive CCT was defined as having a CAC > 100 Agaston units and/or any degree of coronary artery stenosis, up to 24% of the study population were diagnosed with subclinical CAD.^20^ In the past decade, there have been additional studies evaluating the use of CCT in the marathon population. In Germany, Tsiflikas et al.^7^ confirmed a 50% prevalence of subclinical CAD in 50 male marathoners over the age of 45 years with CCT. In the United States, Schwartz et al.^9^ evaluated plaque volume by CCT in 50 male marathoners aged > 45 years in comparison with 23 sedentary age-matched controls. In this study, CAD was detected in 30 (60%) marathoners as compared with 12 (53%) sedentary controls.^9^ Finally, in the Netherlands, Braber et al.^8^ evaluated 318 male marathoners ≥ 45 years of age with normal baseline exercise stress testing for subclinical CAD using CCT. In this study, a total of 184 (59%) individuals were classified as having mild CAD (<5 0% stenosis) and 52 (16%) individuals were diagnosed with >50% stenosis of any coronary vessel on CCT.^8^

In comparison with these contemporary marathon studies where the prevalence of subclinical CAD on CCT was ≥ 50%,^7^, ^8^, ^9^ the MATCH-40 study reported a lower prevalence of only 20%. The discrepancy could be in part due to the different inclusion criteria and lack of a uniform definition of CAD among the 4 marathon CCT studies.^7^, ^8^, ^9^ First, approximately 35% of the MATCH-40 study population comprised female athletes as compared with the other 3 studies, which exclusively included male athletes. In the MATCH-40 study, despite the mean age being similar for both genders, female participants were less likely to present with any degree of silent CAD; if subclinical CAD was detected, only mild CAD (< 50% stenosis) was seen on CCT imaging in 3 women. In addition, given that a significant proportion of women in the MATCH-40 study were either in the premenopausal and perimenopausal age groups (74%), it is plausible that this may be a potential explanation for the lower prevalence of silent CAD in our study population. Second, the MATCH-40 study included a younger cohort with a lower age limit of 40 years, which may further account for a reduced prevalence of CAD. Finally, the previous 3 marathon CCT studies defined CAD using both CAC and/or the presence of coronary artery stenosis, which could account for the higher prevalence of subclinical CAD of >50% in their patient populations. In the MATCH-40 study, although 9 participants demonstrated focal calcification of the coronary arteries, the CCT analysis did not meet predefined criteria for stenosis. Overall, in a population of marathon runners, the prevalence of subclinical CAD using CCT ranged from 20% to 60% between all 4 studies.^7^, ^8^, ^9^

In the evaluation of ischemic heart disease, anatomic delineation of coronary artery stenosis using either CCT or ICA and quantification of functional ischemia by either stress testing, myocardial perfusion imaging (MPI), or SE provide complementary information. Although CCT alone has a high negative predictive value of > 99% for ruling out significant CAD,^21^ this noninvasive imaging modality has limited specificity for identification of severe flow limiting lesions that lead to functional ischemia.^22^ In comparison, functional imaging using SE has excellent specificity for detection of ischemia.^23^ The **Pro**spective **M**ulticenter **I**maging **S**tudy for **E**valuation of Chest Pain (PROMISE) trial recently evaluated 10,003 patients with stable chest pain comparing CCT with standard of care testing of either exercise stress testing, SE, or nuclear stress studies.^24^ The primary outcome included a composite of death, myocardial infarction, or hospitalization for unstable angina.^24^ Patients were randomized into first-line CCT testing (n = 4996) or first-line functional testing (n = 5007).^24^ Of these patients, 4500 anatomic tests and 4602 functional tests were analyzed. Patients were excluded if they received other tests initially, received noncontrast CT only, or did not undergo any diagnostic testing.^24^ Of those who underwent functional testing, 10% underwent exercise testing, 22% underwent SE testing, and 68% underwent nuclear stress studies.^24^ After a median of 26 months of follow-up, the study concluded that CCT provides superior prognostic information compared with functional testing in contemporary patients with stable chest pain with a low burden for myocardial ischemia.^24^ However, little is known about whether both CCT and functional imaging can provide complementary information in the management of asymptomatic marathoners with subclinical CAD.

A number of contemporary marathon studies using CCT to identify subclinical CAD have incorporated the use of functional imaging to a certain degree. In the study by Tsiflikas et al.,^7^ all 50 individuals enrolled in the study had normal exercise stress tests performed at baseline. Among the 24 (48%) individuals over the age of 45 years who were diagnosed with any degree of CAD on CCT, only 1 patient with moderate stenosis on CCT was referred for functional testing with a stress cardiac magnetic resonance imaging; the outcome of the test was not specified. One patient with atypical chest discomfort and high grade stenosis was referred directly to ICA and underwent PCI to the left anterior descending artery without additional functional imaging.^7^ Similarly, in the study by Braber et al.,^8^ where a normal exercise stress test was performed as part of the inclusion criteria, a total of 9 marathon participants were identified with moderate-to-severe CAD with lesions > 50% on CCT. All 9 individuals underwent further follow-up with MPI studies.^8^ Although 7 MPI studies were normal with no evidence of functional ischemia, 2 MPI studies were classified as positive and subsequently referred for PCI.^8^ Unfortunately, the authors did not specify the regions of ischemia on MPI nor the type of PCI completed in these 2 marathoners.^8^ To complement these 2 previous studies, the MATCH-40 study is the first to perform SE in all CCT-positive patients with any degree of CAD stenosis. Among the 13 of 65 (20%) marathoners diagnosed with CAD by CCT in our study cohort, all of the SE were negative for functional ischemia irrespective of the severity of CAD. Although functional assessment of ischemia using either MPI or SE may be considered after the incidental finding of CAD on CCT in the marathon population, SE does not incur additional radiation exposure.

Coronary atherosclerosis continues to be one of the main causes of exercise-related SCD among adult marathon runners ≥ 40 years of age.^2^^,^^25^ As acute coronary syndromes from plaque rupture can often occur in nonangiographic severe lesions, the presence of nonobstructive CAD may be concerning in older athletes participating in marathon races.^20^ Several mechanisms may be involved in this complex process including: (1) regular exhaustive exercise that may induce a rise in vascular oxidative stress; (2) bursts of inflammatory cytokines that may accelerate the atherosclerotic process and impair microvascular integrity; (3) excessive mechanical forces that can cause plaque erosion or fissuring with epicardial thrombus formation; and/or (4) exercise-induced thrombogenicity from increased catecholamine-induced platelet aggregation.^25^, ^26^, ^27^

The role of preventive therapies in this seemingly robust yet vulnerable marathon population remains unknown. In the **S**cottish **Co**mputed **T**omography of the **Heart** Trial, 4126 patients who were evaluated for stable chest pain were randomized to standard of care vs CCT guided and standard of care.^28^ At a median follow-up time of 5 years, the primary composite outcome of death from CAD or nonfatal MI occurred in 2.3% and 3.9% (*P* = 0.004) in the CCT and control arms, respectively, corresponding to a relative risk reduction of 41%.^28^ The **S**cottish **Co**mputed **T**omography of the **Heart** Trial investigators proposed that early and correct diagnosis of CAD entailed subsequent changes in medical management in the CCT-guided arm as a potential cause for the reduction in the primary outcome.^28^ Over the course of a 5-year follow-up, individuals in the CCT arm were more likely to be treated with preventive therapies including lifestyle modification, aspirin, and statin therapy (19.4% vs 13.2%, odds ratio: 1.27).^28^ Similarly, all of the patients identified with CAD by CCT in our MATCH-40 study were started on aspirin and statin therapy for primary prevention.^29^^,^^30^ Whether primary preventative medical therapy with aspirin and lipid-lowering agents may improve positive vessel remodelling in our asymptomatic population of marathon athletes with subclinical CAD will require a longer follow-up.

The MATCH-40 study had a number of limitations. First, as the MATCH-40 study was a cross-sectional study with a relatively small sample size, a larger patient population with longitudinal follow-up using CCT would be required to ascertain the rates of SCD in marathoners ≥ 40 years of age with subclinical CAD. Second, the study protocol did not allow for an age-matched control group, in part due to a small but theoretical risk of radiation exposure from the CCT. Finally, stress echocardiograms were performed only in individuals with subclinical CAD as identified on CCT, which introduces some degree of selection bias.

## Conclusion

The MATCH-40 study is the first to incorporate both CCT and SE in the evaluation of subclinical CAD in the marathon population. Although the overall prevalence of silent CAD was 20%, there was no evidence of functional ischemia detected on SE in this highly competitive cohort. Whether primary preventative medical therapy may improve positive vessel remodelling and decrease the overall burden of SCD in these individuals with subclinical CAD on CCT requires further long-term follow-up.

## Acknowledgements

The authors would like to especially thank the support of the Manitoba 10.13039/100001225Marathon.

## Funding Sources

The MATCH-40 Study was supported from funding from the University Collaborative Research Program from the 10.13039/100010318University of Manitoba, Canada.

## Disclosures

The authors have no conflicts of interest to disclose.
